# Spectral Signatures of Prime Factorization

**DOI:** 10.3390/e28030363

**Published:** 2026-03-23

**Authors:** Giuseppe Mussardo, Andrea Trombettoni

**Affiliations:** 1 SISSA, Via Bonomea 265, 34136 Trieste, Italy; 2 INFN Sezione di Trieste, Via Valerio 2, 34127 Trieste, Italy; 3 Department of Physics, University of Trieste, Strada Costiera 11, 34151 Trieste, Italy

**Keywords:** prime factorization, quantum mechanics, projective measurements

## Abstract

We present a protocol for integer factorization for all integers *N* below a certain cut-off $\Lambda =2^{d}$, grounded in the theory of quantum measurement. In this framework, the factorization of an integer N≤Λ is achieved in a number of steps equal to the total number *I* of primes present in its factorization; explicitly, the procedure consists of a sequence of *I* quantum measurements. The method requires a single-purpose quantum device designed to perform measurements of an observable with a prescribed spectrum. Crucially, the construction of this device involves solving, once and for all, a set of approximately $2^{d}$ differential equations, independently of the specific integer to be factorized. We argue that the initialization task of this device can be efficiently implemented on a quantum computer in *d* steps, thereby decoupling the computational cost of device preparation from the factorization process itself.

## 1. Introduction

The aim of this manuscript is not to present a patent-style proposal for a new device intended to outperform existing, highly optimized classical algorithms for integer factorization (or to enable a practical attack on RSA-2048). Instead, our goal here is simply conceptual and theoretical: to show that quantum mechanics offers a natural and elegant framework in which problems arising in a field as distant from quantum theory as number theory can be formulated and, potentially, solved in an optimal way. The emphasis on the conceptual nature of the idea, rather than on its practical or engineering implementation, is unequivocal. Indeed, any attempt to address infinite families of instances (as the infinite set of integers) necessarily encounters a fundamental mismatch between physics and mathematics: physical systems are unavoidably subject to cutoffs and finite resources, whereas mathematics imposes no such limitations. Consequently, even if a given physical setup were capable of factoring a 100-digit integer, it would always remain true that it does not yet handle a 1000-digit integer, and so on without end. For instance, in what follows, the proposed protocol is endowed with a natural cutoff, defined by an integer $\Lambda =2^{d}$, which must be specified *ab initio* and determines the range of integers that the device is capable of factorizing.

It is worth observing that analogous considerations apply to the well-known Shor algorithm [1,2] for integer factorization. From a practical standpoint, extending its implementation to cryptographically relevant instances such as RSA-2048 would entail the use of quantum error correction at the scale of millions of physical qubits, together with stringent requirements on the mitigation of decoherence-induced errors. Accordingly, to date, experimental validations of the Shor algorithm have been confined to the factorization of comparatively small integers: the factorization of the number 15=3×5, for instance, was done using 7 qubits with an NMR implementation of a quantum computer [3]. Similar demonstrations were performed using photonic [4,5] and solid-state qubits [6], while in 2012, with the *n* qubits control register replaced by a single qubit recycled *n* times, it was achieved the factorization of the integer 21=3×7 [7]. Despite their simplicity, it goes without saying that these examples nevertheless provide a *proof of principle* realization of the algorithm.

Therefore, with the same spirit and the same awareness of the crucial role played by physical constraints in the implementation of a mathematical algorithm such as Shor’s, we present below an alternative different route for integer factorization, based on an algorithm which exploits another genuine quantum property: projective quantum measurements [8,9,10]. As it is well known from quantum mechanics axioms, if a physical system is in a normalised state |ψ〉, a measurement of an observable $\hat{O}$ will yield one eigenvalue α of its spectrum with probability $|\langle \psi |\alpha \rangle {|}^{2}$, where |α〉 is the normalised eigenfunction corresponding to the eigenvalue α

(1) $$\hat{O}\;|\alpha \;\rangle \;=\;\alpha \;|\alpha \;\rangle \;.$$

As a result of the measurement, the system state will change from |ψ〉 to |α〉 (for a recent discussion on quantum meaurement, see [11]). For problems related to number theory, interesting spectra to consider are: (a) the natural numbers, corresponding to the Hamiltonian of an harmonic oscillator [9,10]; (b) the primes [12,13]; and (c) the logarithm of the primes [14,15,16,17,18,19]. Employing such spectra, one may translate number theory problems in quantum physical settings. As an example of this general philosophy, in this paper we show that with a suitable choice of the operator $\hat{O}$ is possible to determine the prime factors of an integer number *N* less than a given cut-off Λ by making a finite set of quantum measurements.

The organization of this article is as follows. After providing a brief overview of classical algorithms for primality testing and integer factorization, we present a quantum algorithm addressing the factorization problem. For expository convenience, the algorithm is initially formulated within the well-established framework of Schrödinger Hamiltonians, wherein the potential terms effectively act as a data repository for integers and prime numbers. Other implementations are, in principle, equally admissible; accordingly, as discussed in the Appendices, we also consider a digital realization of the algorithm. The computational costs of Shor’s and our algorithm are thoroughly discussed in the last part of the paper.

## 2. Classical Primality Tests and Factorization Algorithms

The fundamental theorem of arithmetic states that every natural number *N* greater than 1 is either a prime number $p_{k}$ or can be represented as a product of prime numbers

(2) $$N=p_{a_{1}}^{\alpha_{1}}p_{a_{2}}^{\alpha_{2}}\ldots p_{a_{k}}^{\alpha_{k}}\;\;\;,$$

where $p_{a_{1}}<p_{a_{2}}\ldots <p_{a_{k}}$ are *k* ordered primes and $\alpha_{i}\geq 1$ their multiplicity. Hence, prime numbers may be regarded as the atoms of arithmetic but, in contrast with the finitely many chemical elements, the number of primes is instead infinite, as shown by a classic argument by Euclid dated more than 2000 years ago. The appearance of prime numbers along the integer sequence is completely unpredictable. However, their coarse graining properties, and in particular how many prime numbers there are below any real number *x*, are aspects which can be controlled with remarkable precision. In other words, while there is no known simple function f(n) which gives the *n*-th prime number $p_{n}$ (and the actual determination of prime numbers can only be done by means of the familiar Eratosthenes’s sieve [20,21,22,23,24]), we have instead perfect knowledge of the inverse function π(x) which counts the number of primes below the real number *x* [20,21,22,23,24,25,26,27,28,29,30,31]. Such a function was exactly determined by Riemann (see, for instance [30]): it has a staircase behaviour (since it jumps by 1 each time *x* crosses a prime), but becomes smoother and smoother for increasing values of *x*, and its asymptotic behaviour is constrained by the “Prime Number Theorem” [25] stating that $\lim_{x\to \infty }\frac{\pi (x)\ln x}{x}\;=\;1$. Notice that $p_{n}\;=\;\pi^{-1}\left(n\right)$. Hence, inverting at the lowest order the function π(x), one gets the following scaling law for the *n*-th prime number: $p_{n}≃n\;\log n$.

Let us first discuss the primality test. How can one tell whether an integer *N* is prime? What if the number has hundreds or thousands of digits? This question may seem abstract or irrelevant, but primality tests are performed every time to make online transactions secure. Given an integer *N*, determine whether or not *N* is a prime number constitutes a primality test. The naive way to check the primality of an integer *N* is to divide it by any prime number between 2 and $\sqrt{N}$. Assuming we express the number *N* in binary basis, if *N* is of order $2^{d}$, the number of operations D of this naive primality algorithm scales exponentially with the number of digits, i.e., $D∼2^{d/2}$. Many different algorithms have been proposed, both deterministic or probabilistic nature (see, for instance [32]), to reduce the complexity of the primality test. The final answer was given in 2002 [33] in terms of a deterministic protocol (the AKS test) whose complexity scales as [33] $D_{AKS}∼{\left(\log N\right)}^{7.5}$.

Let us now imagine that the primality test outputs that *N* is not a prime. Then, looking at (2), how do we determine the prime factors $p_{a_{1}}\ldots p_{a_{k}}$ of the integer *N*? This is the question addressed by any factorisation algorithm. Let *N* be a number of *n*-bit digits: currently there is no classical factorization algorithm whose complexity scales $D∼O(n^{\alpha })$ for some constant α. Although neither the existence nor non-existence of such algorithms has been proved, it is generally believed that they do not exist and hence that the problem is not in the class of Polynomial Time Algorithms. If so, then the problem is clearly in class NP, although it is not certain whether it is or is not in the class of NP-complete problems [34,35,36]. The best classical algorithm known is the so-called general number field sieve (GNFS) [35] whose complexity for a number *N* scales as $\exp{\left(\ln N\right)}^{1/3}$.

## 3. The Factorization Algorithm by Quantum Measurements

In the following we discuss how to devise a very efficient algorithm for factorizing integers *N* less than a given cut-off $\Lambda =2^{d}$ using quantum mechanical measurements in a way that the number of steps is finite and does not scale with the *n* digits of *N*. For the implementation of the protocol, it is essential to specify *ab initio* the cutoff Λ and to have an a-priori knowledge of all prime numbers $p_{n}\leq \Lambda$. In our presentation, the operator $\hat{O}$ is the guise of the familiar Hamiltonian $\hat{H}$, but the reader is of course free to substitute the operator $\hat{H}$ with any other hermitian operator with the appropriate spectra. Let us assume that the Hamiltonian $\hat{H}$ is made up of two Hamiltonians ${\hat{H}}_{1}$ and ${\hat{H}}_{2}$ which commute to each other:

(3) $$\hat{H}\;=\;{\hat{H}}_{1}+{\hat{H}}_{2}\;,\;\;\;\;\;\;\;\;\;\;\left[{\hat{H}}_{1},{\hat{H}}_{2}\right]=0\;\;\;.$$

We choose ${\hat{H}}_{1}$ to have Λ eigenvalues given by the logarithms of the primes, where $\Lambda =2^{d}$ is a fixed cut-off

(4) $$E_{n}^{\left(1\right)}\;=\;\log p_{n}\;;\;n=1,2,3,\cdots 2^{d}\;.$$

The corresponding eigenfunctions will be denoted as $|\log p_{n}\rangle$.

On the other hand, the second term ${\hat{H}}_{2}$ in the Hamiltonian $\hat{H}$ is chosen to have the eigenvalues given by the logarithms of the integers, up to the same cut-off Λ, i.e.,

(5) $$E_{m}^{\left(2\right)}=\log m\;;\;m=1,2,3,\ldots 2^{d}\;.$$

The corresponding eigenvectors will be denoted as |logm〉. As discussed below, these Hamiltonians $H_{1}$ and $H_{2}$ could in principle be realised in a laboratory by means of spatial light laser modulators, as it was recently done for a Hamiltonian which had the prime numbers as quantum spectrum [13].

The generic eigenfunctions of $\hat{H}$ are then given by

(6) $$|\log p_{n}\rangle \;|\log m\rangle \;,$$

which correspond to the eigenvalues $E_{n,m}\;\equiv \;\log p_{n}+\log m$.

The factorization problem of a natural number *N* consists of finding the primes entering its decomposition (2). In order to do so, our protocol consists of the following steps.

1. Take initially the logarithm of the number *N* to be factorized, promote it to be an eigenvalue of the Hamiltonian $\hat{H}$ and prepare the initial state, hereafter denoted as |logN〉, which has this energy.
2. For an integer *N*—as the one given in Equation(2)—made of *k* distinct primes, the corresponding energy level of $\hat{H}$ is *k*-fold degenerate, i.e., the degeneracy of the level depends *only* on the number of distinct primes present in *N* and not on their multiplicities. Indeed, logN can be written in the following *k* different ways
  $$\begin{array}{ccc} \log N & \;=\; & \log p_{1}+\log\left(p_{1}^{a_{1}-1}p_{2}^{a_{2}}\ldots p_{k-1}^{a_{k-1}}p_{k}^{a_{k}}\right)\;\equiv \\ & \;\equiv \; & \log p_{1}+\log{\tilde{N}}_{1}\\ \log N & \;=\; & \log p_{2}+\log\left(p_{1}^{a_{1}}p_{2}^{a_{2}-1}..p_{k-1}^{a_{k-1}}p_{k}^{a_{k}}\right)\;\equiv \\ & \;\equiv \; & \log p_{2}+\log{\tilde{N}}_{2}\\ \ldots & \\ \log N & \;=\; & \log p_{k}+\log\left(p_{1}^{a_{1}}p_{2}^{a_{2}}...p_{k-1}^{a_{k-1}}p_{k}^{a_{k}-1}\right)\;\equiv \\ & \;\equiv \; & \log p_{k}+\log{\tilde{N}}_{k} \end{array}$$
  where ${\tilde{N}}_{a}\;\equiv \;N/p_{a}$ is the integer obtained dividing the original number *N* by one of its prime factor $p_{a}$.
3. Hence, the generic state of the *k*-th degenerate manifold with energy logN (as before, here simply denoted as |logN〉) admits the expansion
  (7) $$|\log N\rangle \;=\;\sum_{a=1}^{k}c_{a}|\log p_{a}\rangle |\log N_{a}\rangle \;,$$
  with $\sum_{a=1}^{k}{\left|c_{a}\right|}^{2}=1$. For a generic state, we can assume that all coefficients of this expansion are different from zero. Their values are actually not essential for the running of the algorithm, so one may assume to be randomly distributed, as would occur in the case of a random initial state preparation.
4. After the state (7) is prepared, measure ${\hat{H}}_{1}$. With all coefficients $c_{a}$ different from zero, the output will be the logarithm of one of the primes present in *N*, say $\log p_{b}$, with probability $|c_{b}{|}^{2}$.
5. Once the result of this measurement is known, divide the original number *N* by the prime $p_{b}$ identified by the output of the ${\hat{H}}_{1}$ measurement. In this way, one obtains the lower integer ${\tilde{N}}_{b}=N/p_{b}$. Then, start over, taking ${\tilde{N}}_{b}$ as the integer to be factorized. The procedure will halt after a number of iterations *l* equal to the total number of primes present in *N*, i.e.,
  (8) $$I\;=\;\sum_{l=1}^{k}\alpha_{l}\;\;\;.$$

Notice that the number of quantum measurements can be made equal to the number of distinct factors substituting the previous point 5 with this new one.

6. Once the result of this measurement is known, divide the original number *N* by the prime $p_{b}$ identified by the output of the ${\hat{H}}_{1}$ measurement. In this way one obtains the lower integer $N/p_{b}$. Use a classical computer to continue to divide for $p_{b}$ till the obtained number is not longer divisible for $p_{b}$. In this way, one obtains the multiplicity $\alpha_{b}$ associated to the factor $p_{b}$. Then, start the procedure again, taking ${\tilde{N}}_{b}=N/p_{b}^{\alpha_{b}}$ as the integer to be factorized.

**Figure 1:**
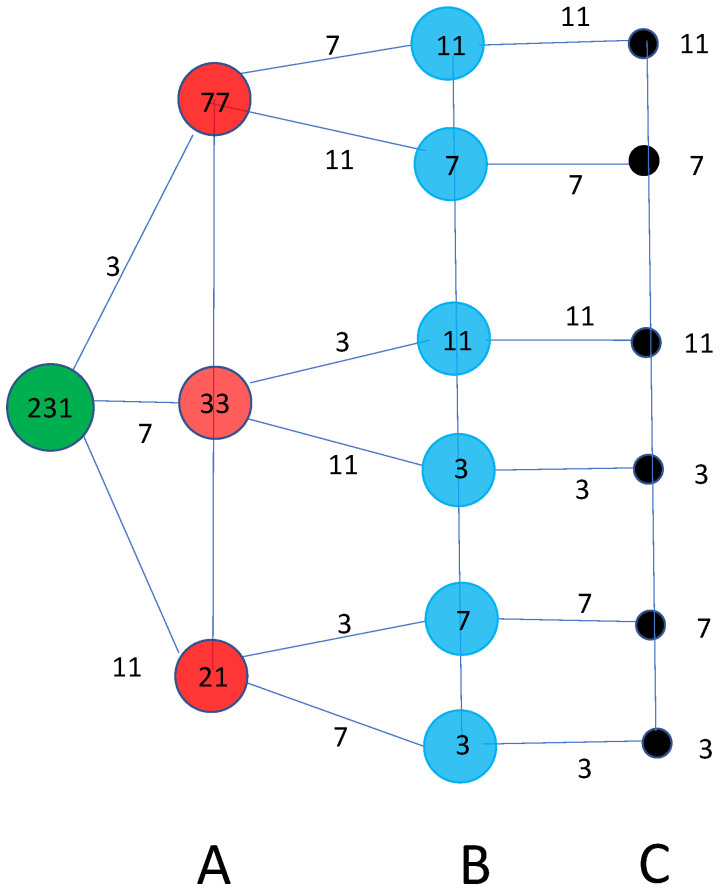
The graph associated with the various quantum measurements of ${\hat{H}}_{1}$ which pin down the different prime factors of *N*. In the example shown in the figure, the number to factorize is N=231=3×7×11. For simplicity, along the links of the graph, we indicate the primes (rather than their logarithm) which are the outputs of the three stages A, B, C measurements of ${\hat{H}}_{1}$. One gets a complete factorisation independently of the path associated with the chain of ${\hat{H}}_{1}$ measurements.

Three significant remarks are in order:

- A primality test can be immediately implemented by performing a single quantum measurement of $\hat{H_{1}}$ on the initial state |logN〉. If the system collapses (remains) in itself, *N* is a prime.
- If the multiplicity of the last factor, say $p_{k}$, to be factorized is 1, then the last operation with the measurement of ${\hat{H}}_{1}$ actually amounts to apply the identity operator. If the multiplicity is different from 1, i.e., $\alpha_{k}>1$, then $\alpha_{k}$ subsequent quantum measurements of ${\hat{H}}_{1}$ will produce each time with probability 1 the same eigenstate $|\log p_{k}\rangle$.
- The successful implementation of the algorithm is guaranteed independently of the ${\hat{H}}_{1}$ outputs obtained at the various stages of the algorithm. It is an *all roads lead to Rome* procedure. As shown in Figure 1, there are possible branches resulting from successive measurements of ${\hat{H}}_{1}$. Imagine, for instance, that we want to factorize the number N=231, whose prime decomposition is given by N=3×7×11. As a result of the first measurement, we could have one of three possible outputs: log3, log7 or log11.
  1. If the first output is log3, the next integer to be factorized is ${\tilde{N}}_{3}=77$ and the next measurement of $H_{1}$ starting from the (log) of this number can give, as output, either log7 or log11. Once this last measurement is made, the next number is uniquely determined, thus, arriving to the complete factorization of the number N=231. It is easy to see that the same conclusion will be reached if there is a different initial output.
  2. If the first output is log7, the next integer to be factorized is ${\tilde{N}}_{7}=33$ and the next measurement of $H_{1}$ starting from the (log) of this number, can give, as output, either log3 or log11. Once this last measurement is made, the next number is uniquely determined, thus arriving to the complete factorization of the number N=231.
  3. If the first output is log11, the next integer to be factorized is ${\tilde{N}}_{11}=21$ and the next measurement of $H_{1}$ starting from the (log) of this number can give, as output, either log3 or log7. Once this last measurement is made, the next number is uniquely determined, thus, arriving at the complete factorization of the number N=231.

The key feature of this algorithm is the projective quantum measurements of hermitian operators, such as the Hamiltonians we have employed. This leads to an algorithm with the least possible number of operations, equal to *k*, relative to the factorization of an integer made of *k* primes, see below for a discussion of this point.

Given that quantum mechanics is based on linear algebra while the mathematical problem relative to factorization involves products, Hamiltonians with logarithm of the primes as spectrum are very natural to study and have been also used before [15,16,17,18]. In more detail, a thermally isolated non-interacting Bose gas loaded in a one-dimensional potential with logarithmic energy eigenvalues was discussed in [15], where asymptotic formulas for factorising products of different primes were provided. Time-dependent perturbation in (possibly multiple copies of) a potential having logarithms of the primes as eigenvalues was instead proposed in [16,17,18], where the number *N* to be factorized is encoded in the frequency of a sinusoidally modulated interaction acting on the ground-state of the potential. In this approach, if the integer is a product of *k* primes, one needs to prepare an ensemble of *k* identical systems each with an energy spectrum given by the logarithm of the primes [18]. Recently, a paper discussed the possible use of a Hamiltonian having as eigenvalues the logarithm of the primes for search algorithm [37].

Our approach differs from the one discussed in [18] for two key features: first, the presence of ${\hat{H}}_{2}$ in our Hamiltonian and, secondly, the use of quantum measurements. Employing our Hamiltonian, made of a piece relative to the logarithm *and* another one relative to the logarithm of the integers, it is possible to factorize an integer *N* without knowing a-priori the number *k* of distinct primes (and their multicplicities) this number *N* is made of. Concerning the quantum measurement of ${\hat{H}}_{1}$, one could use the von Neumann scheme [38], which consists of setting up a coupling with a test particle of the form $\hat{p}\;\hat{O}$, where $\hat{p}$ is the momentum operator of the test particle and $\hat{O}$ the operator to be measured. As a matter of fact, the explicit implementation of the von Neumann measurement scheme constitutes a problem of intrinsic interest, warranting a separate and detailed investigation, which will be presented in forthcoming work [39]. For the purposes of the present analysis, it is sufficient to assume that the operator ${\hat{H}}_{1}$ is measurable, as is the case for any quantum observable.

## 4. Quantum Potentials

Let us now discuss a possible implementation of the algorithm described in the previous section. Essentially, we require a convenient database of natural numbers and prime numbers below a given cutoff Λ. One possible approach is to encode these numbers as spectra of Schrödinger Hamiltonians. This is similar to what has been done recently [13] for a quantum potential of the primes. Hence, we will deal with Hamiltonians of a particle of mass *m* of the form $\hat{H}\;=p^{2}/2m+V\left(x\right)$ with their eigenvalues determined by the Schrödinger equation $\hat{H}\psi_{n}=e_{n}\psi_{n}$.

A potential $V_{N}\left(x\right)$ entering a Schrödinger Hamiltonian with a finite and assigned number of eigenvalues $\left\{e_{0},e_{1},...,e_{M}\right\}$ can be constructed using methods of Supersymmetric Quantum Mechanics (SQM) [40,41,42]. The protocol is as follows:

1. First of all, we subtract from all the eigenvalues $e_{n}$ the highest one $e_{N}$, so that the new set of numbers $\left\{{\tilde{E}}_{n}\right\}$
  (9) $${\tilde{E}}_{k}\;=\;e_{N-k}-e_{N},\;k=0,1,\ldots ,N,$$
  will be considered as the new spectrum. The ${\tilde{E}}_{k}$’s are of course the (negative) gaps computed from the *highest* eigenvalue $e_{N}$. Notice that, consistently, they are enumerated starting from the top to the bottom, so ${\tilde{E}}_{0}=0$, ${\tilde{E}}_{1}$ is the first gap, ${\tilde{E}}_{2}$ the second gap, and so on. A potential V(x) where its only eigenvalue is ${\tilde{E}}_{0}=0$ is of course $V_{0}\left(x\right)=0$. This potential is used as input for the Riccati equation for the super-potential $W_{1}\left(x\right)$
  (10) $$W_{1}^{\prime }\left(x\right)-W_{1}^{2}\left(x\right)+V_{0}\left(x\right)\;=\;{\tilde{E}}_{1}$$
  with boundary condition $W_{1}\left(0\right)=0$.
2. Once such a function W(x) has been obtained, one can construct another potential $V_{1}\left(x\right)$ as
  (11) $$V_{1}\left(x\right)\;=\;2{\tilde{E}}_{1}+2W_{1}^{2}\left(x\right)-V_{0}\left(x\right)\;\;\;.$$
  This potential is then substituted into Equation (10) (i.e., $V_{1}\left(x\right)\to V_{0}\left(x\right)$, substituting also ${\tilde{E}}_{1}\to {\tilde{E}}_{2}$), so that one has a differential equation for another super-potential $W_{2}\left(x\right)$
  (12) $$W_{2}^{\prime }\left(x\right)-W_{2}^{2}\left(x\right)+V_{1}\left(x\right)\;=\;{\tilde{E}}_{2}\;\;\;,$$
3. Proceeding iteratively in this way, one has a recursive sequence of differential equations
  (13) $$\begin{array}{c} W_{k}^{\prime }\left(x\right)-W_{k}^{2}\left(x\right)+V_{k}\left(x\right)\;=\;{\tilde{E}}_{k}\;\;\;,\\ V_{k}\left(x\right)=2{\tilde{E}}_{k}+2W_{k}^{2}\left(x\right)-V_{k-1}\left(x\right) \end{array}$$
  with all of them being solved with the boundary condition $W_{k}\left(0\right)=0$, which ensures that the final potential $V\left(x\right)=V_{N}\left(x\right)$ is an even function. This recursive system is continued until all the gaps have been taken into account. Hence, solving (in general numerically) the differential equations of Equation (13), one arrives to the Hamiltonian which has *exactly* the spectrum $\left\{e_{n}\right\}$
  (14) $$H\;=\;-\frac{d^{2}}{dx^{2}}+V_{N}\left(x\right)+e_{N}\;\;\;,$$

These are indeed the theoretical steps which lead to the potential $V_{N}\left(x\right)$ with *exactly* the first *M* prime numbers [13,42] or the logarithm of the first *M* primes [14].

**Figure 2:**
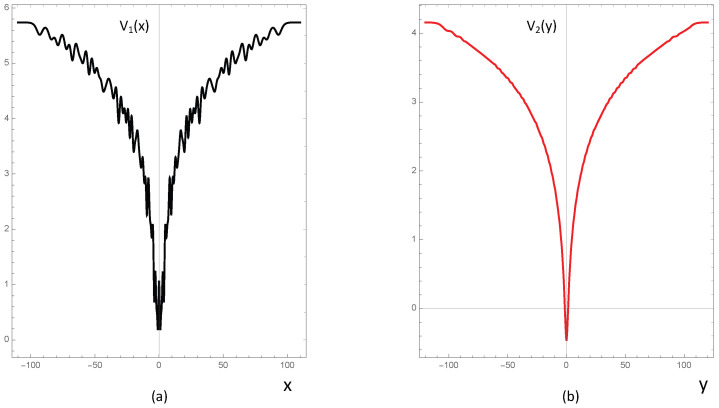
Plots of the potentials $V_{1}\left(x\right)$ (**a**) and $V_{2}\left(y\right)$ (**b**) relative to $\Lambda =64=2^{5}$ energy levels given respectively by the logarithm of the primes, $\log p_{k}$, and the logarithm of the integers, logm.

To conclude the implementation of our algorithm, let us then define the potentials $V_{1}\left(x\right)$ and $V_{2}\left(y\right)$ entering the two Schrödinger Hamiltonians ${\hat{H}}_{1}$ and ${\hat{H}}_{2}$ of the previous section (it is worth recalling that both potentials $V_{1}\left(x\right)$ and $V_{2}\left(y\right)$ can be also constructed in a semi-classical approximation [12] for *all* primes and integers.)

(15) $${\hat{H}}_{1}\left(x\right)\;=\;\frac{p_{x}^{2}}{2m}+V_{1}\left(x\right),\;\;\;\;\;\;\;{\hat{H}}_{2}\left(y\right)\;=\;\frac{p_{y}^{2}}{2m}+V_{2}\left(y\right),$$

where $p_{x}$ and $p_{y}$ are the *x* and *y* components of its momentum, and such ${\hat{H}}_{1}$ (${\hat{H}}_{2}$) has as eigenvalues the logarithm of the first $2^{d}$ primes (respectively, the logarithm of the first $2^{d}$ integers). The plot of $V_{1}\left(x\right)$ and $V_{2}\left(y\right)$ in a specific case is shown in Figure 2. Incidentally, if the Goldbach conjecture were true, taking the same Hamiltonian ${\hat{H}}_{1}$ in both the *x* and *y* direction, i.e., $H={\hat{H}}_{1}\left(x\right)+{\hat{H}}_{1}\left(y\right)$, the spectrum of H would be given by the logarithms of *all* even numbers.

## 5. Discussion and Conclusions

Once we have the two Hamiltonians ${\hat{H}}_{1}$ and ${\hat{H}}_{2}$, we can build up the Hamiltonian $\hat{H}={\hat{H}}_{1}+{\hat{H}}_{2}$ and proceed to factorize the integers *N* below the cut-off Λ through a sequence of *k* measurements of ${\hat{H}}_{1}$, where *k* is the number of different prime factors the number *N* is made of. As an example, if *N* is a product of three primes, only three quantum measurements are needed.

One can show that *k* is clearly the lower bound of operations which are needed to factorize an integer, independently from quantum mechanics. Indeed, suppose that the integer *N* has the form (2) and that one is given the information that $p_{1},\ldots ,p_{k}$ are its factors. To verify it, one would divide by one of them, say $p_{1}$, and continue by it till it is possible ($\alpha_{1}+1$ divisions), and so on, for a total of $\sum_{i=1}^{k}\alpha_{i}+k$ divisions. If one is also provided the information about the multiplicities, then only *k* divisions are needed. In the latter case, the last division is trivial.

The procedure proposed here saturates such lower bounds. Indeed, from the first quantum measurement one gets one of the factors, and then divides *N* (e.g., using a classical computer) by the latter till the obtained number is no longer divisible. Iterating this way one needs *k* quantum measurements (the last, *k*-th, being equal to the identity) and $\sum_{i=1}^{k}\alpha_{i}$ divisions on the classical computer. One sees that the factorization based on quantum measurements saturates the lower bound, whether the multiplicities are given or they are not.

Underlying this procedure, however, is the nontrivial problem of constructing a data repository for integers and prime numbers below a prescribed cutoff $\Lambda =2^{d}$. If this information is encoded in the potentials of two Schrödinger Hamiltonians, the task amounts to solving a system of Λ coupled Riccati equations. This issue is more appropriately regarded as a hardware-level rather than a software-level problem, in the sense that it must be addressed once and for all in order to enable access to the factorization of all integers N≤Λ. A noteworthy observation is that the computational complexity of this construction scales as logΛ=d, as argued in [43]. Indeed, it was discussed in [43] that a system of *M*, possibly non-linear, differential equations of the form

(16) $$\frac{d\vec{W}}{dx}+f\left(W\right)\cdot \vec{W}=\vec{b}\;,$$

where f is a M×M matrix and $\vec{b}$ is a constant vector, can be solved on a quantum computer in logM number of steps. Namely, there is an exponential speed up which turns our problem to adjust an exponential number $2^{d}$ of eigenvalues in each of the Hamiltonians $H_{1}$ and $H_{2}$ in terms of only *d* computational steps.

(17) $$f_{ab}\;=\;\left\{\begin{array}{cccc} 0 & , & \;\; & a>b\\ -W_{a} & , & \;\; & a=b\\ {\left(-1\right)}^{a}2W_{b} & , & \;\; & a<b \end{array}\right.$$

and

(18) $$b_{k}={\tilde{E}}_{k}+2\sum_{i=1}^{k-1}{\left(-1\right)}^{i}{\tilde{E}}_{k-i}\;.$$

Hence the exponential growth of complexity of our procedure which arises at its hardware level can be cured using an ordinary quantum computer.

In summary, the factorization procedure proposed in this paper for all integers *N* less than a given cut-off $\Lambda =2^{d}$ consists in two distinct parts: (i) the implementation of an integer and prime data repository which consists in solving $2^{d}$ differential equations, a task which an universal quantum computer can solve in *d* number of steps, i.e., in. polynomial time; (ii) a series of *k* quantum measurements, where *k* is the number of different primes entering the prime decomposition of the integer *N*.

## Data Availability

The original contributions presented in this study are included in the article material. Further inquiries can be directed to the corresponding author.
